# P-190. Tick and Flea-Borne Disease Over the Last Twenty Years in the Military

**DOI:** 10.1093/ofid/ofaf695.413

**Published:** 2026-01-11

**Authors:** Lauren Sweet, Elena Crouch, John Kiley

**Affiliations:** Brooke Army Medical Center, San Antonio, TX; Brooke Army Medical Center, San Antonio, TX; BAMC, San Antonio, Texas

## Abstract

**Background:**

The burden of emerging tick and flea-borne disease in the United States (US) is increasing, but the true prevalence remains undefined. Given the military’s frequent field training, these infections have a potential impact on force readiness. Studies evaluating more diverse geographic ranges within the US remain to be conducted, particularly in the military. Here, the Defense Medical Surveillance System (DMSS)—an archive of all clinical data of the DOD serum repository—is evaluated for prevalence and characteristics of various rickettsial diseases diagnosed within the military system.

Table 1

**Figure:**
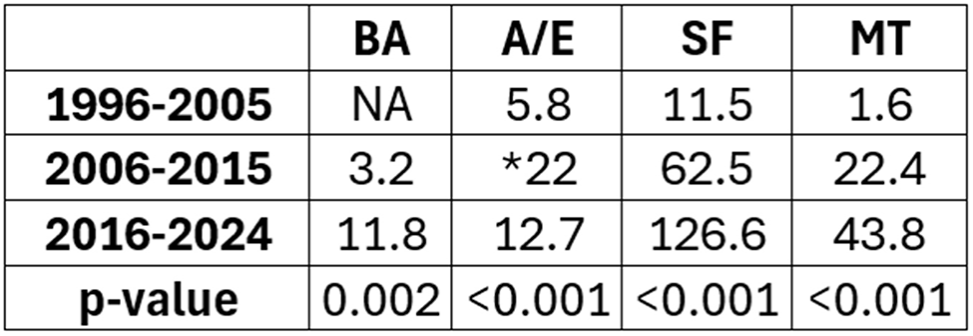

Mean cases per year of each disease by decade. SF: Spotted fever; MT: Murine typhus; A/E: Anaplasmosis/Ehrlichiosis; BA: Babesiosis. Note: laboratory data only became available in 2007. *The decade of 2006-2015 had significantly more cases of A/E when compared to decades before or after.

**Figure 1:**
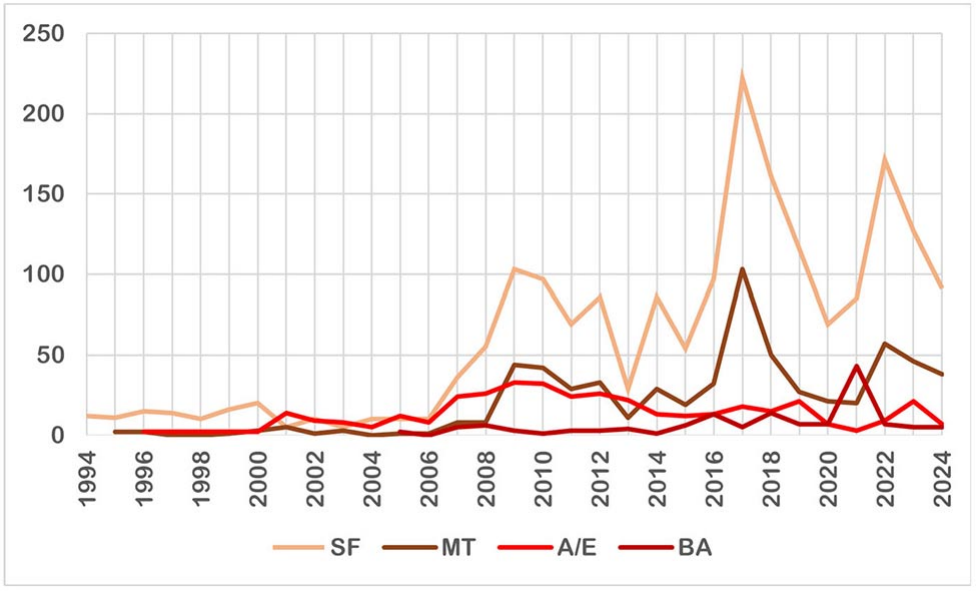
Number of cases per each disease from 1994-2024. SF: Spotted fever; MT: Murine typhus; A/E: Anaplasmosis/Ehrlichiosis; BA: Babesiosis. Note: laboratory data only became available in 2007.

**Methods:**

A retrospective descriptive study of epidemiological data in the DMSS was performed. The DMSS was queried for all confirmed and suspected cases of Murine Typhus (MT), Spotted Fever (SF), Ehrlichiosis/Anaplasmosis (A/E) and Babesiosis (BA) between 1994 and 2024. Confirmed (reportable medical event or positive lab result) and suspected (1 inpatient or 2 outpatient visits within 60 days with a disease-associated ICD-code) cases as defined by the DMSS were included.

**Figure 2:**
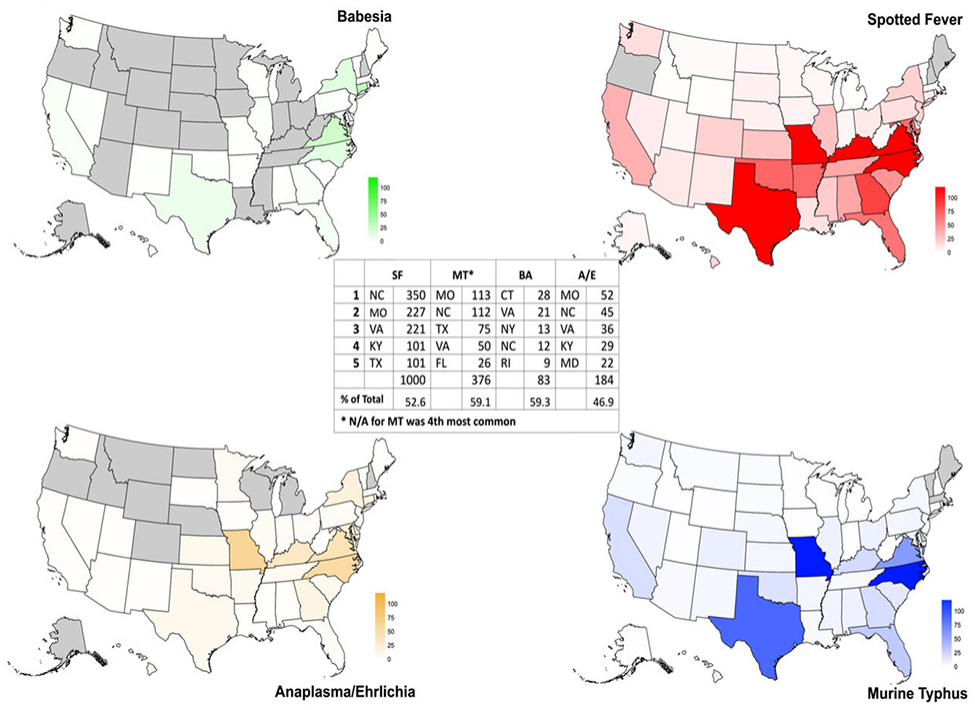
Geographic distribution of cases since 1994, and the number of cases in the top 5 states, with a percent of total cases. SF: Spotted fever; MT: Murine typhus; A/E: Anaplasmosis/Ehrlichiosis; BA: Babesiosis; N/A: not specified

**Results:**

Overall, 1902 cases of SF (since 1994), 636 cases of MT (since 1995), 140 cases of BA (since 2005), and 392 cases of A/E (since 1996) were identified. There were significant increases in BA, SF and MT across the decades of 1995-2005, 2006-2015, 2016-2024 (Fig. 1). A/E also saw a significant increase from 2006-2015. When comparing 2016-2024 to 2006-2015, mean cases of BA nearly quadrupled (3.2 to 11.8 cases/year), SF doubled (62.5 to 126.6 cases/year) and MT doubled (22.4 cases to 43.8 cases/year) (Table 1). The top 5 states made up roughly 50% of all cases (Fig. 2). White, male and age < 35 was associated with more diagnoses.

**Conclusion:**

This inquiry into the DMSS data confirms several aspects of these TFDs—most notably their year-over-year increases. Lab data became available only in 2007, which may drive some of the growth. The data also supports that a handful of states present an opportunity for potential study and focused intervention. While BA, A/E and SF are nationally reportable diseases, MT is not, which makes this data useful, in that it suggests there is potentially under-recognized burden in states such as Missouri. Understanding the changing geographic distribution of TFD is paramount as vectors expand their home ranges

**Disclosures:**

All Authors: No reported disclosures

